# The Fate of Acetic Acid during Glucose Co-Metabolism by the Spoilage Yeast *Zygosaccharomyces bailii*

**DOI:** 10.1371/journal.pone.0052402

**Published:** 2012-12-28

**Authors:** Fernando Rodrigues, Maria João Sousa, Paula Ludovico, Helena Santos, Manuela Côrte-Real, Cecília Leão

**Affiliations:** 1 Life and Health Sciences Research Institute (ICVS), School of Health Sciences, University of Minho, Braga, Portugal; 2 ICVS/3B's - PT Government Associate Laboratory, Braga/Guimarães, Portugal; 3 Centre of Molecular and Environmental Biology (CBMA)/Department of Biology, University of Minho, Braga, Portugal; 4 Instituto de Tecnologia Química e Biológica, New University of Lisbon, Oeiras, Portugal; Université de Nice-CNRS, France

## Abstract

*Zygosaccharomyces bailii* is one of the most widely represented spoilage yeast species, being able to metabolise acetic acid in the presence of glucose. To clarify whether simultaneous utilisation of the two substrates affects growth efficiency, we examined growth in single- and mixed-substrate cultures with glucose and acetic acid. Our findings indicate that the biomass yield in the first phase of growth is the result of the weighted sum of the respective biomass yields on single-substrate medium, supporting the conclusion that biomass yield on each substrate is not affected by the presence of the other at pH 3.0 and 5.0, at least for the substrate concentrations examined. *In vivo* ^13^C-NMR spectroscopy studies showed that the gluconeogenic pathway is not operational and that [2−^13^C]acetate is metabolised via the Krebs cycle leading to the production of glutamate labelled on C_2_, C_3_ and C_4_. The incorporation of [U-^14^C]acetate in the cellular constituents resulted mainly in the labelling of the protein and lipid pools 51.5% and 31.5%, respectively. Overall, our data establish that glucose is metabolised primarily through the glycolytic pathway, and acetic acid is used as an additional source of acetyl-CoA both for lipid synthesis and the Krebs cycle. This study provides useful clues for the design of new strategies aimed at overcoming yeast spoilage in acidic, sugar-containing food environments. Moreover, the elucidation of the molecular basis underlying the resistance phenotype of *Z. bailii* to acetic acid will have a potential impact on the improvement of the performance of *S. cerevisiae* industrial strains often exposed to acetic acid stress conditions, such as in wine and bioethanol production.

## Introduction

Acetic acid is a weak carboxylic acid that acts as an important environmental stressor negatively affecting the yeast metabolic activity and ultimately leading to fermentation arrest and cell death [1]. Spoilage yeasts are, however, able to circumvent the acid induced cytotoxicity, and hence may survive and ferment under such conditions causing serious economic losses. *Zygosaccharomyces bailii* has been described as one of the most important food and beverages spoilage yeast species characteristic of acidic products. The responses of *Z. bailii* cells to weak carboxylic acids have been mainly studied in media with acetic acid and glucose, at low pH [2]–[8]. Acetic acid may induce stimulatory effects on growth and fermentative metabolism of *Z. bailii*, with consequences at earlier spoilage ability even at refrigeration temperature [9]. Previous studies have shown that respiration and fermentation of glucose by *Z. bailii* were exponentially inhibited by acetic acid, though the inhibition was much less pronounced than in *Saccharomyces cerevisiae* [10]. Moreover, in contrast to *S. cerevisiae*, the inhibitory effects of acetic acid were not significantly potentiated by ethanol, providing a plausible explanation for the ability of *Z. bailii* to re-ferment sugars in wines. Consistently with the acid resistance phenotype of *Z. bailii* the concentration of acetic acid able to induce cell death was much higher in this species than in *S. cerevisiae* [3], [5].

In turn, the high resistance of *Z. bailii* to acetic acid has also been related with its capacity to transport and metabolise this weak acid in the presence of glucose [7], [8], contrary to most of the yeast described, namely *S. cerevisiae*, *Candida utilis*, *Torulaspora delbrueckii* and *Dekkera anomala*, where active transport of acetate is inducible and subjected to glucose repression [11]–[14]. However, in chemostat cultures of *S. cerevisiae* grown in mixtures of glucose and acetic acid, the glucose concentration can be so low that the cells are no longer repressed and can metabolize acetate concomitantly with glucose [15]. Some commercial *S. cerevisiae* wine strains are also able to consume acetic acid in the presence of glucose [16]. This behaviour resembles that found in *Z. bailii* ISA 1307 grown in a medium containing glucose and acetic acid at pH 5.0. Indeed, under this growth condition *Z. bailii* ISA 1307 displays a biphasic growth, where the first phase is associated with simultaneous consumption of both substrates, and the second one with the utilization of the remaining acid. Such ability has been associated with the high activity not only of acetic acid transport but also of the acetyl-CoA synthetase [8], [17].

The aim of the current study was to identify the biochemical pathways associated with acetic acid utilization during co-metabolism with glucose by *Z. bailii* ISA1307 that could, somehow, direct the design of new strategies to overcome yeast spoilage in acidic environments. Thus, the metabolic fate of acetic acid in *Z. bailii* was evaluated during alcoholic fermentation, under aerobic conditions, using a combination of complementary approaches to elucidate if yeast growth efficiency was affected and if both substrates, glucose and acetic acid, were used as carbon and/or energy sources. To achieve these purposes, the biomass yields on single-substrates (glucose or acetic acid) were determined and correlated with those obtained for mixed-substrate cultures (glucose plus acetic acid). In vivo, carbon-13 (^13^C) nuclear magnetic resonance spectroscopy and ^14^C incorporation experiments were used to identify the pathways of acetic acid metabolism in whole cells of *Z. bailii*. Our findings showed that the yeast growth efficiency on mixed-substrate medium with glucose and acetic acid was not affected and that the acid acts as an additional source of acetyl-CoA being redirected to Krebs cycle and lipid biosynthesis. Therefore, it appears that *Z. bailii* benefits from an additional energy source of respiratory metabolism of acetic acid, even under glucose-fermentative conditions, turning the co-metabolism of the acid-stressor agent into a spoilage advantage.

## Materials and Methods

### Microorganism and Maintenance

The yeast *Zygosaccharomyces bailii* ISA 1307 originally isolated from a continuous production plant of sparkling wine was used. The cultures were maintained on YEPD agar slants containing glucose (2%, w/v), peptone (1%, w/v), yeast extract (0.5%, w/v) and agar (2%, w/v).

### Growth Conditions

*Zygosaccharomyces bailii* ISA 1307 was grown in a synthetic mineral medium with vitamins [18] supplemented with different carbon and energy sources (see results), at pH 3.0 and 5.0. The cultures were performed in flasks containing a 2∶1 ratio of gas to liquid phase in an orbital shaker (160 r.p.m.) at 26°C. Growth was monitored through the increase in OD measured at 640 nm.

### Estimation of Cell Dry Weight and Biomass Yield

Dry weight of cells (dry wt) in culture samples (100 ml) were determined using acetate cellulose filters (pore size 0.45 µm). After removal of the medium by filtration, the filters were washed with 200 ml of ice-cold distilled water and dried overnight at 80°C. In the case of single-substrate medium the samples were taken immediately after exhaustion of glucose or acetic acid in the culture medium. The experimental biomass yields in the mixed-substrate cultures (pH 3.0 and 5.0), were estimated at the end of the first growth phase, corresponding to the simultaneous utilisation of the two substrates.

### Estimation of Glucose and Acetic Acid Concentrations

Glucose and acetic acid concentrations in the media were assayed by high-performance liquid chromatography, using a Refractive Index detector and a Polyspher OA KC (Merck) column. Arabinose was used as an internal standard.

### Preparation of Cells for *in vivo* NMR Experiments

For *in vivo* NMR experiments, yeast cells were harvested in the mid-exponential phase of growth (mixed-substrate medium, pH 5.0), washed twice with ice-cold distilled water and finally with phosphate buffer 50 mM (pH 5.0). The cells were resuspended in the same buffer, to a final concentration of 4–5 mg dry weight ml^−1^ and transferred to a 10-mm NMR tube. At time designated zero, [2−^13^C]acetate and unlabelled glucose were added at final concentrations of 83 mM and 63 mM, respectively. The kinetics of substrate consumption and product formation was monitored *in vivo* by ^13^C-NMR, at 26°C, under aerobic conditions achieved by using an air-lift system [19].

### NMR Spectroscopy

^13^C-NMR spectra were recorded at 125.77 MHz on a Bruker DRX500 spectrometer with a quadruple nucleus probe head. Spectra were acquired with proton broad-band decoupling, a repetition delay of 1.7 s and a pulse width of 15 µs, corresponding to 90° flip angle. The ^2^H resonance of ^2^H_2_O was used to lock the field and for shimming.

### *In vivo* ^14^C Incorporation Experiments and Fractionation of Cellular Compounds

Yeast cells were grown in 100 ml of culture medium (pH 5.0) containing 2% (w/v) glucose and 0.2% (v/v) [U-^14^C]acetate (1.9 kBq/ml) and harvested after 17 h (middle of the first exponential growth phase). In these experiments, a higher concentration of glucose was used in order to increase the biomass concentration in the sample harvested. This concentration of glucose did not affect the utilisation of acetate (results not shown); at the sampling time used, about 25% of the acid had been metabolised, while glucose remained in the medium at concentrations above 1% (w/v).

For the fractionation of cellular compounds the modified procedure of Sutherland and Wilkinson was used [20]. The harvested cells were filtered on nitrocellulose membrane filters and washed three times with NaCl solution (0.9%, w/v), and twice with cold TCA (10%, v/v). The washed-cell residue was refluxed for 5 min in 1.5 ml of methanol. After cooling to room temperature, the refluxed mixture was stirred for 15 min with 3.0 ml of chloroform and centrifuged at 15,000× *g*. The residue was further extracted once with 2.0 ml of methanol-chloroform (1∶3, by volume) and five times with 2.0 ml each of chloroform. These extracts were combined as the crude lipid fraction. The defatted residue was extracted twice by 30-min stirring with 2.0 ml of TCA (5%, v/v) at 90°C. Hot TCA extracts were combined as the crude nucleic acid fraction. The remaining residue was stirred with 1.5 ml of water and 1.5 ml of 90% phenol at 65°C for 15 min. Centrifugation resulted in two layers; polysaccharides partitioned preferentially into the water layer while proteins were extracted into the phenol layer. An adequate volume of each cellular fraction was mixed with the scintillation fluid OptiPhase Hisafe II (LKB Scintillation Products) and the radioactivity was measured in a Packard TriCarb 2200 CA liquid scintillation counter. Proportionality between the sample volume of the different fractions and radioactivity counts was confirmed.

### Chemicals

Radioactively labelled acetic acid was obtained from Radiochemical Center (Amersham) and had the following specific activity: [U-^14^C]acetic acid, sodium salt, 2.2×10^9 ^Bq mmol^−1^. [2−^13^C]acetate, sodium salt was obtained from Sigma. All other chemicals were reagent grade and were obtained from commercial sources.

### Reproducibility of the Results

Data are reported as the mean ± standard error of the mean of at least three independent repetitions of each assay. The statistical analyses were performed using the SPSS program version 19 and GraphPad software.

## Results

### Specific Growth Rates and Biomass Yields on Single and Mixed-substrate Medium with Glucose and Acetic acid

**Figure 1:**
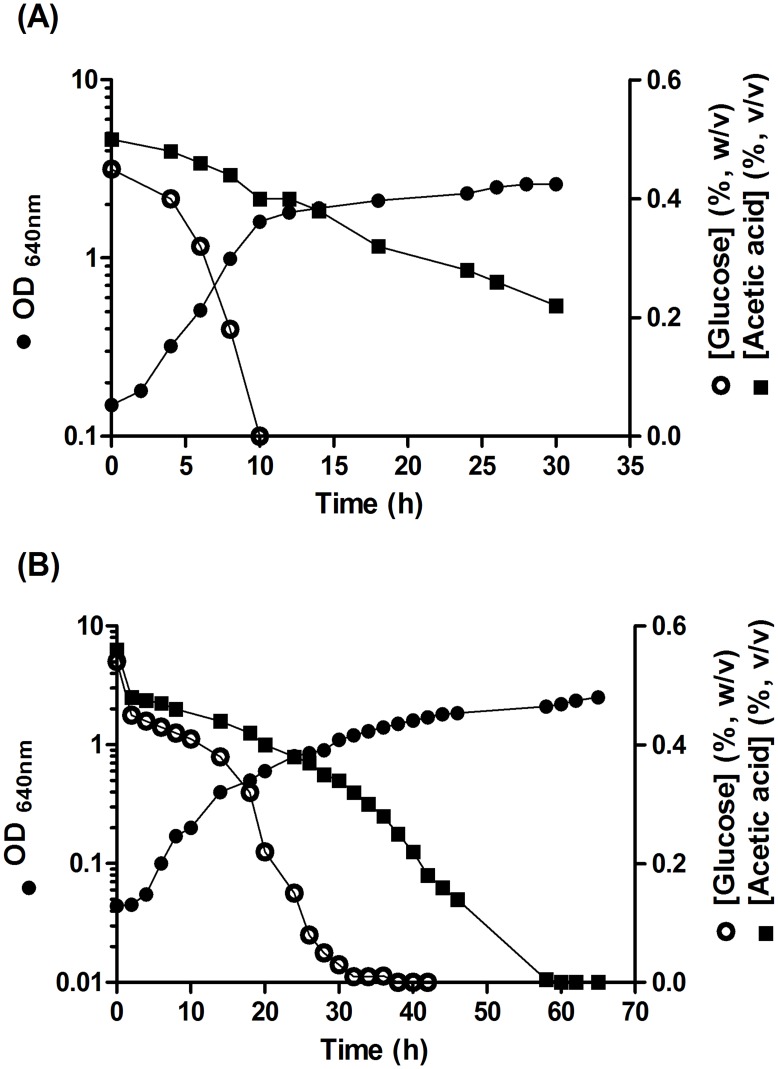
Growth of *Z. bailii* ISA 1307 at pH 5.0 (A) and pH 3.0 (B) in a medium containing glucose (0.5%, w/v) and acetic acid (0.5%, v/v).

To determine whether simultaneous utilisation of glucose and acetic acid affects growth efficiency, we examined growth on single- and mixed-substrate cultures with glucose and acetic acid. As reported previously [8], growth of *Zygosacharomyces bailii* ISA 1307 in a medium containing a mixture of glucose (0.5%, w/v) and acetic acid (0.5% v/v) at pH 3.0 or 5.0, was biphasic, the acid and the sugar being simultaneously utilised during the first growth phase (Figure 1). The specific growth rates and biomass yields on single-substrates (glucose or acetic acid) and mixed-substrate cultures (glucose plus acetic acid) in the first growth phase were determined (Table 1). For cells grown in glucose plus acetic acid at pH 3.0, in contrast with pH 5.0, a decrease of the specific growth rate (about 23%) was observed when compared with cells grown in glucose as only carbon and energy source. The observed decrease at pH 3.0 means that acetic acid is affecting growth efficiency, expressed by the specific growth rate, and may be explained by the increase in the maintenance energy required to overcome intracellular acidification associated with the acid dissociation in the cytosol at this pH, when compared with pH 5.0. This effect is also evidenced when comparing the values of specific growth rate and growth yields in acetic acid single medium at pH 3.0 with those obtained at pH 5.0 (Table 1).

**Table 1:** Specific growth rates (h^−1^) and biomass yields of *Z. bailii* ISA 1307 (Yx/s, [g dry wt. g^−1^ carbon]) in medium with glucose (0.5%, w/v) and/or acetic acid (0.5%, v/v) at pH 3.0 and 5.0.

Substrates	µ(h^−1^)		Yx/s(g dry wt. g^−1^ carbon)	
	pH 3.0	pH 5.0	pH 3.0	pH 5.0
**Glucose**	0.212±0.018	0.203±0.016	0.499±0.050	0.521±0.023
**Acetic acid**	0.088±0.006	0.125±0.009	0.634±0.058	0.888±0.076
**Glucose and** **acetic acid**	0.164±0.014	0.268±0.022	0.518±0.049	0.586±0.036
**Glucose and** **acetic acid** *	NA	NA	0.537	0.592

The theoretical biomass yields values in mixed medium calculated by the equation 1.

* theoretical biomass yields values calculated by the equation 1; NA – not applicable.

When simultaneous utilisation of different carbon and energy sources occurs and efficiency of growth is not affected, the biomass yields on single and mixed-substrates are correlated according to the following equation [21]:
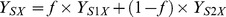
(1)where, Y_sx_ is the biomass yield on substrate carbon (g biomass [g substrate carbon]^−1^) of the mixed-substrate culture, Y_s1x_ and Y_s2x_ are the biomass yields on acetic acid carbon and glucose carbon, respectively. The value of *f* is the weighted fraction of acetic acid carbon consumed during mixed-substrate utilisation.

Table 1 shows the experimental values for the biomass yields obtained in *Z. bailii* when growing in single substrate media with glucose (0.5%, w/v) or acetic acid (0.5%, v/v) at pH 3.0 and 5.0. These experimental values were used for estimating the theoretical values for biomass yield in mixed-substrate media according to equation 1. The experimental biomass yields in mixed-substrate media were also determined in the first growth phase (Table 1). The experimental and theoretical values obtained for biomass yields in mixed-substrate media were identical both for pH 3.0 and 5.0 (Table 1), suggesting that both substrates were used as carbon and energy sources and that growth efficiency on each substrate, expressed as the biomass yield, was not affected by the presence of the other.

Thus, in the next sections particular attention was directed to the elucidation of the metabolic fate of acetic acid during co-metabolism with glucose.

### *In vivo* Analysis of Acetic Acid Co-metabolism by ^13^C-NMR Spectroscopy

**Figure 2:**
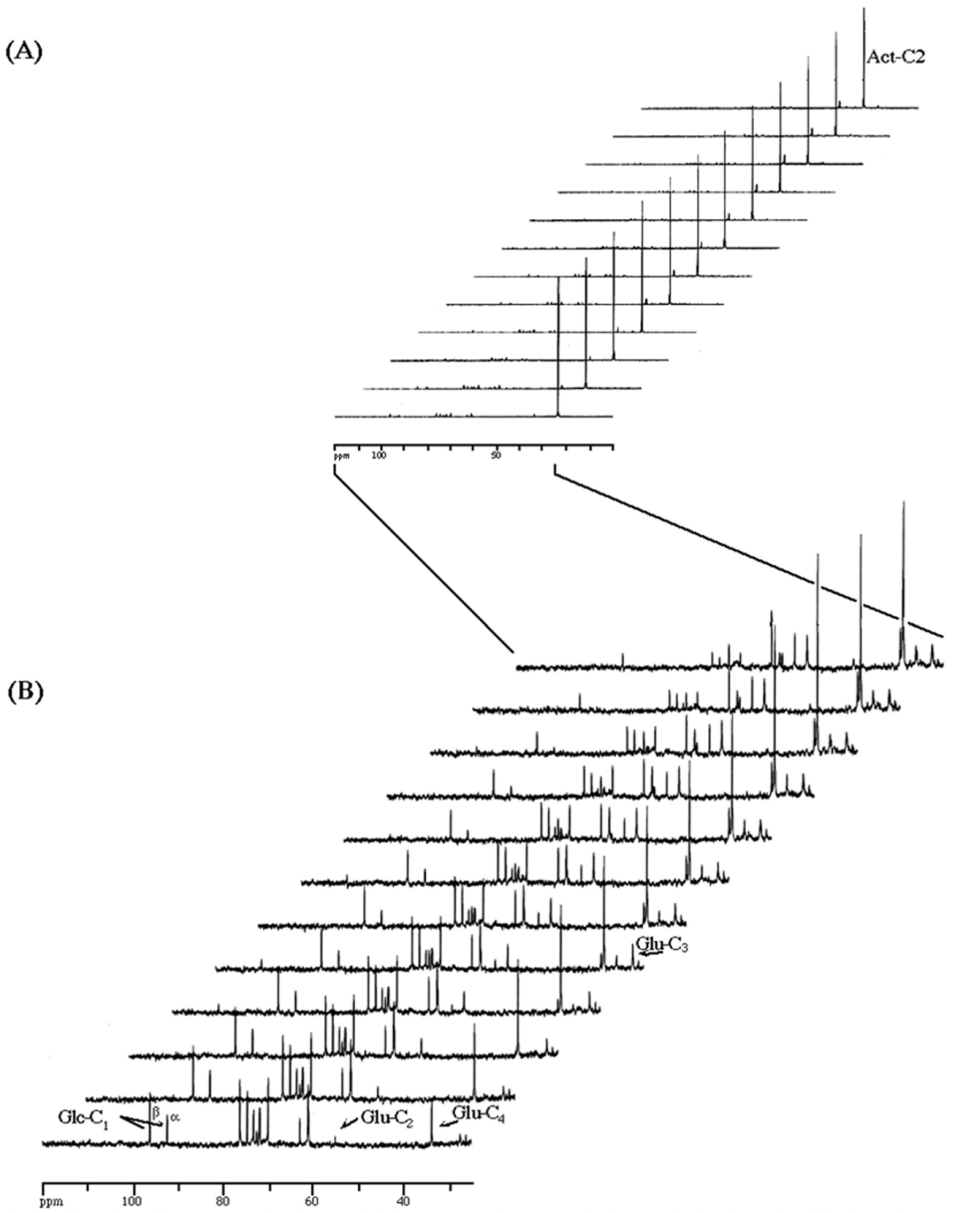
*In vivo* ^13^C-NMR spectra of [2−^13^C]acetate metabolism and unlabelled glucose by resting cells of *Z. bailii*, in aerobic conditions. [2-^13^C]acetate (83 mM) and unlabelled glucose (63 mM) were added to a cell suspension (4–5 mg dry weight ml^-1^). ^13^C-NMR spectra (2.5 min each) were acquired sequentially. An expansion of (A) in the spectral region 25–120 ppm is shown in (B). Symbols: Act-C_2_ is [2–^13^C]acetate; Glu-C_2_, C_3_ and C_4_ are the resonances due to carbon atoms 2, 3, and 4, respectively, in glutamate; Glc-C_1_ (α,β) are the resonances due to carbon 1 in the two anomers of glucose (natural abundance).

Resting cells of *Z. bailii*, harvested from the middle of the first exponential growth phase in mixed medium with glucose and acetic acid, pH 5.0, were used to follow the metabolic fate of acetic acid. The cells were incubated with [2−^13^C]acetate, in the presence of unlabelled glucose and used to follow the kinetics of acid utilisation by *in vivo* ^13^C-NMR spectroscopy. The results obtained (Figure 2), besides confirming the simultaneous consumption of glucose and acetic acid, allowed tracing the metabolic fate of acetic acid. Thus, in an expansion of Figure 2A it was possible to follow the low-intensity resonances due to the carbon 13 natural abundance in the two anomers of glucose, as well as the resonances due to C_2_, C_3_ and C_4_ of glutamate (Figure 2B). In the first spectrum, glutamate was labelled primarily on C_4_ and to a lower extent on C_2_ and C_3_, as expected for the metabolism of [2-^13^C]acetyl-CoA through the Krebs cycle. Furthermore, the last spectrum in the sequence clearly showed the typical multiplet pattern in the glutamate resonances originated by several turns of the cycle, as previously described [22]–[24], indicating that in the presence of glucose, acetic acid was metabolised through the Krebs cycle.

Also, the absence of labelled trehalose or glycerol from [2-^13^C]acetate indicates the lack of activity of the gluconeogenic pathway. This view was further supported by the lack of detection, either *in vivo* or in perchloric acid extracts, of labelled alanine (results not shown) and is consistent with the absence of *in vitro* activity of phosphoenolpyruvate carboxykinase [8]. Moreover, no labelled aspartate was detected pointing to a small ratio between labelled and unlabelled oxaloacetate, and hence to a high flux of pyruvate through pyruvate carboxylase (Pyc), that culminates in a high dilution of the label in this intermediary.

### *In vivo* Analysis of Acetic Acid Co-metabolism by ^14^C Incorporation

The *in vivo* ^13^C-NMR studies described above did not allow concluding whether acetic acid, in the presence of glucose, was channelled to lipid biosynthesis. For such purpose, cells of *Z. bailii* were harvested from the middle of the first exponential growth phase of cultures in mixed-substrate medium with glucose and [U-^14^C]acetate at pH 5.0. In these samples the incorporation of [U-^14^C]acetate in the different cellular constituents was measured, as described in experimental procedures. The results presented in Table 2, showed that 51.5% of the radioactivity detected was incorporated into the protein fraction. This agrees with the observation of labelled glutamate from labelled acetate by *in vivo* ^13^C-NMR spectroscopy and reinforces the view that the acid was metabolised through the Krebs cycle producing α-ketoglutarate and oxaloacetate, two amino acid precursors. On the other hand, about 31.5% of [U-^14^C]acetate incorporated was found in the lipidic fraction, indicating that the acid metabolism also contributed to lipid biosynthesis. The label was also present in the nucleic acid fraction, accounting to only 6.3% of the total labelled compounds. The presence of radioactivity in the nucleic acid fraction could be explained by the involvement among others of glutamine, glycine and aspartate on the biosynthetic pathways of purines and pyrimidines. However, only the amino groups of glutamine are involved in those reactions, therefore the amino acid, even with labelled carbon, will not contribute for the labelling of nucleic acids. Glycine could not be labelled since gluconeogenesis is not active under the growth conditions used [8]. Thus, only aspartate could contribute to the labelling of nucleic acids.

**Table 2:** Incorporation of [U-^14^C]acetate, in the presence of glucose, into cellular constituents of *Z. bailii* ISA 1307 growing in medium with glucose (2%, w/v) and acetic acid (0.2%, v/v) at pH 5.0.

	% of total labelling†
Proteins	51.5
Lipids	31.5
Low molecular mass compounds	10.6
Nucleic acids	6.3
Polysaccharides	0.1

† The values presented are the average of two independent experiments, with a variation lower than 5%.

## Discussion

As it has been described so far in other yeasts, namely *Saccharomyces cerevisiae* and *Torulaspora delbrueckii* [11], [12], high concentrations of glucose in the medium generally repress the utilisation of less energetic substrates, the so called glucose repression effect [25]. This regulation pattern by glucose was not found in *Z. bailii*, which is able to use acetic acid as a carbon and energy source, both alone and in the presence of glucose [8]. *Schizosaccharomyces pombe* metabolises acetic acid in the presence of glucose but can not use the acid or other 2C compounds as the only carbon and energy source [20] due to the lack of isocitrate lyase and malate synthetase [26]. Recently, some commercial *S. cerevisiae* wine strains have been described as being able to consume acetic acid in the presence of glucose [16]. However, such strains have only been studied under a practical point of view and the physiological traits underlying their ability to co-metabolize acetic acid and glucose remain to be elucidated.

The results here presented show that in *Z. bailii* grown on mixed-substrate media with glucose and acetic acid, the biomass yield during the simultaneous utilization of both substrates at pH 3.0 and 5.0 is the result of the weighted sum of the respective biomass yields on single-substrate medium, indicating that the biomass yield on each substrate is not affected by the presence of the other. In contrast to that observed for biomass yields, at pH 3.0 the presence of acetic acid in mixed medium negatively affects the specific growth rate when compared with that on single glucose medium. Cell response to stress caused by the intracellular acidification following acetic acid dissociation at the intracellular pH, most relevant at pH 3.0, will likely increase the maintenance energy required for pH homeostasis. In acetic acid single medium such increase in maintenance energy, has a negative impact on the growth rate and biomass yields as we can observe by comparing pH 3.0 with pH 5.0. This same pH effect is observed on the mixed medium. On the other hand, while comparing growth in glucose single medium with mixed medium at pH 3.0, the increase in the maintenance energy is reflected on a decrease of the specific growth rate, but the same effect is not evidenced on the biomass yield. This may be explained by the fact that the biomass yield on acetic acid single medium is higher than on glucose, and so the weighted sum of the biomass yields, which is the value for the biomass yield on mixed substrate medium, will be higher than the biomass yield on single glucose medium.

According to the known pathways in *S. cerevisiae*, the utilisation of [2-^13^C]acetate should result in a first step in [2−^13^C]acetyl-CoA through the activity of acetyl-CoA synthetase. Two genes have been described that code for acetyl-CoA synthetase in *S. cerevisiae, ACS1* and *ACS2* [27], [28]. The *ACS2* gene is constitutively expressed and is essential for growth on glucose, since it codes for the cytosolic acetyl-CoA synthetase izoenzyme that maintains the cytosolic acetyl-CoA pool needed for lipid synthesis, although not allowing acetate metabolism in the presence of glucose [8], [27], [28]. *Z. bailii* genome also encodes such a constitutive enzyme (*ZbACS2*) although with higher specific activity than in *S. cerevisiae* [8], [17], [27]. The identification of *ZbACS2* was consistent with previous detection of acetyl-CoA syntethase activity in cells grown in glucose plus acetic acid (first phase) though with a lower activity than that in acetic acid-grown cells [8], [17]. This physiological feature together with the presence of an acetate carrier subjected to control by the intracellular acid concentration warrants a metabolic flux compatible with the maintenance of an intracellular acetic acid concentration below the values above which toxic effects may occur. The relevance of *ZbACS2* to the acid resistance of *Z. bailii* was confirmed by the higher resistance of *S. cerevisiae* expressing *ZbACS2* to acidic environments with sugars. Indeed these *S. cerevisiae* cells exhibited an increased resistance to acetic acid expressed by a reduction in the lag phase time [17].

Several works have been published concerning the determination in *S. cerevisiae* of the metabolic fluxes through the Krebs and glyoxylate cycles by ^13^C-NMR [22]–[24], [29]. In these studies it was possible to assume that all acetyl-CoA entering these cycles was labelled. In the present work, concerning glucose-acetic acid grown cells of *Z. bailii*, the pool of acetyl-CoA was not completely labelled. The acetyl-CoA besides being produced through acetic acid (labelled) metabolism could also be produced from glucose (unlabelled) by the pyruvate dehydrogenase (PDH) complex or by the so called PDH bypass [30]. Furthermore, [2−^13^C]acetyl-CoA once entering either the Krebs or glyoxylate cycles gives rise to ^13^C-glutamate [22]. The contribution of the glyoxylate cycle to the metabolism of acetyl-CoA can be ruled out since it has been shown that isocitrate lyase and malate syntethase activities under the experimental conditions used are absent [8]. In turn, the detection of typical multiplet pattern in the glutamate ressonances supports the interpretation that in the presence of glucose, acetic acid was metabolised through the Krebs cycle.

Also the non detection of labelled aspartate reflects a high glycolytic flux that enables its production mainly through unlabelled oxaloacetate generated by pyruvate carboxylase (Pyc)-mediated carboxylation of pyruvate. This result is in accordance with the observed increase of the biomass yield in the mixed-substrate cultures since Pyc, the only anaplerotic enzyme active under these conditions, allows the replenishing of the Krebs cycle with biosynthetic precursors and the production of more precursors for biosynthesis during co-metabolism of acetic acid and glucose.

The ^14^C-acetic acid incorporation experiments confirmed that this compound was mainly channelled for protein and lipid synthesis and in a lesser extension was also redirected to nucleic acid biosynthesis. ^14^C-acetic acid incorporation in lipids can occur in the cytoplasm with the involvement of acetyl-CoA metabolism through the malonyl-CoA pathway as described for *S. cerevisiae.* The label was also present in the nucleic acid fraction, accounting to only 6.3% of the total labelled compounds. As referred in results section, only aspartate appears to contribute to the labelling of nucleic acids. The observed low level of ^14^C nucleic acid being related to the small amount of labelled aspartate corroborates this hypothesis. This result is consistent with the inability of detection of labelled aspartate by *in vivo* ^13^C-NMR, and is in agreement with earlier results with glucose- and galactose-grown cells of *S. cerevisiae* [31]. Under such conditions, a high pool of oxaloacetate (via Pyc) was observed comparatively to that of acetyl-CoA (via pyruvate dehydrogenase, Pdh) after addition of pyruvate.

**Figure 3:**
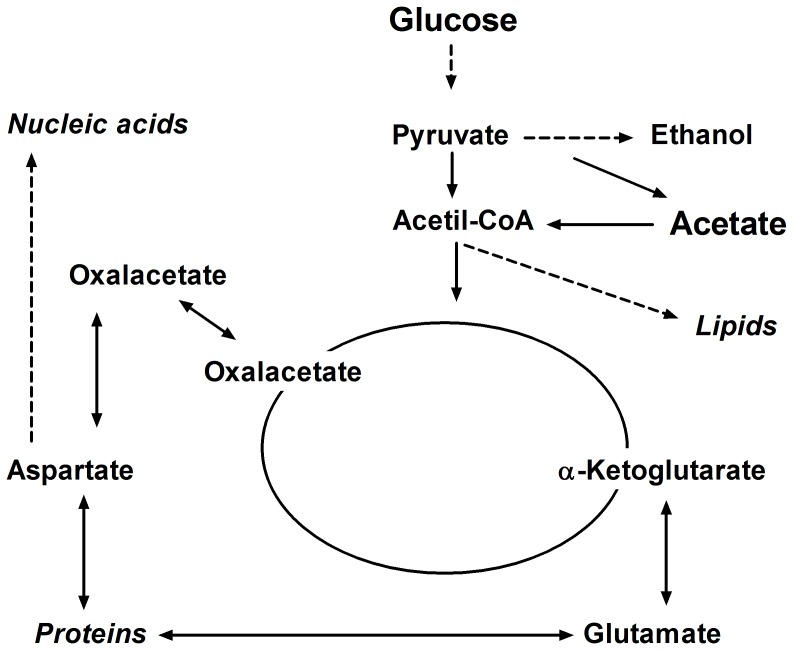
Major metabolic pathways involved in the simultaneous utilisation of glucose and acetic acid in *Z. bailii* ISA 1307.

As a whole, our studies concerning the simultaneous utilization of glucose and acetic acid by *Z. bailii* indicate that this species displays a peculiar pattern for the regulation of acetic acid metabolism, unusual in yeasts and different from that described for most *S. cerevisiae* strains. A scheme for such metabolic model is proposed in Figure 3 and the following points should be emphasised: (i) in mixed-substrate media both glucose and acetic acid are used as carbon and energy sources; (ii) gluconeogenesis is not operational in resting cells of glucose-acetic acid grown cells (first phase of growth); (iii) the anaplerotic step in the replenishment of the Krebs cycle, catalysed by Pyc, is enhanced in the presence of acetic acid to cope with the additional provision of acetyl-CoA generated from acetic acid metabolism; (iv) acetic acid is converted into acetyl-CoA which functions as an additional source both for the Krebs cycle and for the synthesis of lipids.

The data now presented further contribute to understanding the high resistance exhibited by *Z. bailii* to acidic environments with sugars. The ability of this species to metabolise acetic acid in the presence of glucose is an advantage for such environments since it results in a decrease of the intracellular pool of acetate, and the co-metabolism allows the production of further energy that can be spent on cellular maintenance. It would be interesting to genetically manipulate *Z. bailii* to abolish its ability to co-metabolise acetic acid and glucose and prove that such strains would be more sensitive to the presence of this preservative. However, data from *S. cerevisiae* has pointed out that mutants in *ACS2* are unable to support growth on glucose [28] due to their inability to replenish the needed acetyl-CoA pool for lipid synthesis. Therefore, such intervention in *Z. bailii* would, most probably, result in an identical phenotype.

In summary, our findings show that *Z. bailii* in comparison with less acid tolerant species, benefits from an additional energy source due to the respiratory metabolism of acetic acid, even under glucose-fermentative conditions. Therefore, under a practical point of view, our results further reinforce acetyl-CoA syntethase a potential molecular target in the design of new strategies to overcome yeast spoilage in acidic environments with sugars.

Moreover, the elucidation of the molecular basis underlying the resistance phenotype of *Z. bailii* to acetic acid may have a relevant impact on the improvement of the performance of *S. cerevisiae* industrial strains often exposed to acetic acid stress conditions. In fact, acetic acid is a sub-product alcoholic fermentation and is also present in lignocellulosic acid hydrolysates used for bioethanol production. Therefore, acetic acid has a negative impact on yeast performance, restraining the production efficiency of wine, bioethanol or of products obtained by heterologous expression with engineered yeast cells under fermentative conditions [32], [33], [34]. Several unsuccessful attempts have been made to produce *S. cerevisiae* mutants able to metabolise significant amounts of acetic acid in the presence of glucose namely, by UV radiation and overexpression of *ZbACS2* (our unpublished data). Although the reasons underlying such unexpected phenotype are unknown, one can hypothesize, based on the results obtained in this study, that they might be related to the low respiratory capacity of *S. cerevisiae* cells in the presence of glucose due to catabolic repression. It will therefore be pertinent in the future to genetically and metabolically characterize the *S. cerevisiae* wine strains described as being able to consume acetic acid in the presence of glucose [16].

## References

[1] Sousa MJ, Ludovico P, Rodrigues F, Leao C, Corte-Real M (2012) Stress and Cell Death in Yeast Induced by Acetic Acid. In: Bubulya P, editors. Cell Metabolism - Cell Homeostasis and Stress Response. Available from: http://www.intechopen.com/books/cell-metabolism-cell-homeostasis-and-stress-response/stress-and-cell-death-in-yeast-induced-by-acetic-acid.

[2] Arneborg N, Jespersen L, Jakobsen M (2000) Individual cells of *Saccharomyces cerevisiae* and *Zygosaccharomyces bailii* exhibit different short-term intracellular pH responses to acetic acid. Archives of Microbiology 174: 125–128. 10985752 10.1007/s002030000185

[3] Fernandes L, Corte-Real M, Leao C (1999) A peculiar behaviour for cell death induced by weak carboxylic acids in the wine spoilage yeast *Zygosaccharomyces bailii* . Letters in Applied Microbiology 28: 345–349.

[4] Guerreiro JF, Mira NP, Sa-Correia I (2012) Adaptive response to acetic acid in the highly resistant yeast species *Zygosaccharomyces bailii* revealed by quantitative proteomics. Proteomics 12: 2303–2318. 22685079 10.1002/pmic.201100457

[5] Ludovico P, Sansonetty F, Silva MT, Corte-Real M (2003) Acetic acid induces a programmed cell death process in the food spoilage yeast *Zygosaccharomyces bailii* . FEMS Yeast Research 3: 449–450. 10.1016/s1567-1356(02)00166-6 12702251

[6] Prudencio C, Sansonetty F, Corte-Real M (1998) Flow cytometric assessment of cell structural and functional changes induced by acetic acid in the yeasts *Zygosaccharomyces bailii* and *Saccharomyces cerevisiae* . Cytometry 31: 307–313. 9551607 10.1002/(sici)1097-0320(19980401)31:4<307::aid-cyto11>3.0.co;2-u

[7] Sousa MJ, Miranda L, CorteReal M, Leao C (1996) Transport of acetic acid in *Zygosaccharomyces bailii*: Effects of ethanol and their implications on the resistance of the yeast to acidic environments. Applied and Environmental Microbiology 62: 3152–3157. 8795203 10.1128/aem.62.9.3152-3157.1996 PMC168109

[8] Sousa MJ, Rodrigues F, Corte-Real M, Leao C (1998) Mechanisms underlying the transport and intracellular metabolism of acetic acid in the presence of glucose in the yeast *Zygosaccharomyces bailii* . Microbiology-Uk 144: 665–670. 10.1099/00221287-144-3-665 9580346

[9] Dang TD, Vermeulen A, Ragaert P, Devlieghere F (2009) A peculiar stimulatory effect of acetic and lactic acid on growth and fermentative metabolism of Z*ygosaccharomyces bailii* . Food Microbiol 26: 320–327. 19269576 10.1016/j.fm.2008.12.002

[10] Fernandes L, CorteReal M, Loureiro V, Loureiro Dias MC, Leao C (1997) Glucose respiration and fermentation in *Zygosaccharomyces bailii* and *Saccharomyces cerevisiae* express different sensitivity patterns to ethanol and acetic acid. Letters in Applied Microbiology 25: 249–253. 9351272 10.1046/j.1472-765x.1997.00214.x

[11] Casal M, Leao C (1995) Utilization of Short-Chain Monocarboxylic Acids by the yeast *Torulaspora delbrueckii* - specificity of the transport-systems and their regulation. Biochimica et Biophysica Acta-Molecular Cell Research 1267: 122–130. 10.1016/0167-4889(95)00067-3 7612664

[12] Cassio F, Leao C, Vanuden N (1987) Transport of lactate and other short-chain monocarboxylates in the yeast *Saccharomyces cerevisiae* . Applied and Environmental Microbiology 53: 509–513. 3034152 10.1128/aem.53.3.509-513.1987 PMC203697

[13] Cassio F, Leao C (1993) A comparative study on the transport of L-malic acid and other short-chain carboxylic acids in the yeast *Candida utilis*: evidence for a general organic acid permease. Yeast 9: 743–752. 8368008 10.1002/yea.320090708

[14] Geros H, Cassio F, Leao C (2000) Utilization and transport of acetic acid in *Dekkera anomala* and their implications on the survival of the yeast in acidic environments. Journal of Food Protection 63: 96–101. 10643776 10.4315/0362-028x-63.1.96

[15] dos Santos MM, Gombert AK, Christensen B, Olsson L, Nielsen J (2003) Identification of in vivo enzyme activities in the cometabolism of glucose and acetate by *Saccharomyces cerevisiae* by using 13C-labeled substrates. Eukaryot Cell 2: 599–608. 12796305 10.1128/EC.2.3.599-608.2003 PMC161459

[16] Vilela-Moura A, Schuller D, Mendes-Faia A, Corte-Real M (2008) Reduction of volatile acidity of wines by selected yeast strains. Applied Microbiology and Biotechnology 80: 881–890. 18677471 10.1007/s00253-008-1616-x

[17] Rodrigues F, Zeeman AM, Cardoso H, Sousa MJ, Steensma HY, et al (2004) Isolation of an acetyl-CoA synthetase gene (*ZbACS2*) from *Zygosaccharomyces bailii* . Yeast 21: 325–331. 15042592 10.1002/yea.1081

[18] van Uden N (1967) Transport-limited fermentation and growth of *Saccharomyces cerevisiae* and its competitive inhibition. Arch Mikrobiol 58: 155–168. 5600788 10.1007/BF00406676

[19] Santos H, Turner DL (1986) Characterization of the improved sensitivity obtained using a flow method for oxygenating and mixing cell-suspensions in NMR. Journal of Magnetic Resonance 68: 345–349.

[20] Tsai CS, Mitton KP, Johnson BF (1989) Acetate assimilation by the fission yeast, *Schizosaccharomyces pombe* . Biochem Cell Biol 67: 464–467. 2590529 10.1139/o89-073

[21] de Jong-Gubbels P, Vanrolleghem P, Heijnen S, van Dijken JP, Pronk JT (1995) Regulation of carbon metabolism in chemostat cultures of *Saccharomyces cerevisiae* grown on mixtures of glucose and ethanol. Yeast 11: 407–418. 7597844 10.1002/yea.320110503

[22] Tran-Dinh S, Herve M, Wietzerbin J (1991) Determination of flux through different metabolite pathways in *Saccharomyces cerevisiae* by ^1^H-NMR and ^13^C-NMR spectroscopy. Eur J Biochem 201: 715–721. 1682149 10.1111/j.1432-1033.1991.tb16333.x

[23] Tran-Dinh S, Beganton F, Nguyen TT, Bouet F, Herve M (1996) Mathematical model for evaluating the Krebs cycle flux with non-constant glutamate-pool size by ^13^C-NMR spectroscopy. Evidence for the existence of two types of Krebs cycles in cells. Eur J Biochem 242: 220–227. 8973636 10.1111/j.1432-1033.1996.0220r.x

[24] Tran-Dinh S, Hoerter JA, Mateo P, Bouet F, Herve M (1997) A simple mathematical model and practical approach for evaluating citric acid cycle fluxes in perfused rat hearts by ^13^C-NMR and ^1^H-NMR spectroscopy. Eur J Biochem 245: 497–504. 9151985 10.1111/j.1432-1033.1997.t01-2-00497.x

[25] Ronne H (1995) Glucose repression in fungi. Trends Genet 11: 12–17. 7900189 10.1016/s0168-9525(00)88980-5

[26] Fiechter A, Fuhrmann GF, Kappeli O (1981) Regulation of glucose metabolism in growing yeast cells. Adv Microb Physiol 22: 123–183. 7036694 10.1016/s0065-2911(08)60327-6

[27] De VC, Burckert N, Barth G, Neuhaus JM, Boller T, et al (1992) Cloning and disruption of a gene required for growth on acetate but not on ethanol: the acetyl-coenzyme A synthetase gene of *Saccharomyces cerevisiae* . Yeast 8: 1043–1051. 1363452 10.1002/yea.320081207

[28] Van den Berg MA, Steensma HY (1995) *ACS2*, a *Saccharomyces cerevisiae* gene encoding acetyl-coenzyme A synthetase, essential for growth on glucose. Eur J Biochem 231: 704–713. 7649171 10.1111/j.1432-1033.1995.tb20751.x

[29] Jones JG, Sherry AD, Jeffrey FM, Storey CJ, Malloy CR (1993) Sources of acetyl-CoA entering the tricarboxylic acid cycle as determined by analysis of succinate ^13^C isotopomers. Biochemistry 32: 12240–12244. 8218301 10.1021/bi00096a037

[30] Pronk JT, Wenzel TJ, Luttik MA, Klaassen CC, Scheffers W, et al (1994) Energetic aspects of glucose metabolism in a pyruvate-dehydrogenase-negative mutant of *Saccharomyces cerevisiae* . Microbiology 140 (Pt 3): 601–610. 10.1099/00221287-140-3-601 8012582

[31] Sumegi B, McCammon MT, Sherry AD, Keys DA, McAlister-Henn L, et al (1992) Metabolism of [3–^13^C]pyruvate in TCA cycle mutants of yeast. Biochemistry 31: 8720–8725. 1390657 10.1021/bi00152a006

[32] Lee YY, Iyer P, Torget RW (1999) Dilute-acid hydrolysis of lignocellulosic biomass. Adv Biochem Eng Biotechnol 65: 93–115.

[33] Maiorella B, Blanch HW, Wilke CR (1983) By-product inhibition effects on ethanolic fermentation by *Saccharomyces cerevisiae* . Biotechnol Bioeng 25: 103–121. 18548541 10.1002/bit.260250109

[34] Palmqvist E, Hahn-Hägerdal B (2000) Fermentation of lignocellulosic hydrolysates. I: inhibition and detoxification. Bioresour Technol 74: 17–24.

